# ADVANCE CARE PLANNING FIDELITY TOOL IN THE CONTEXT OF COGNITIVE IMPAIRMENT: THE SHARE TRIAL

**DOI:** 10.1093/geroni/igad104.0041

**Published:** 2023-12-21

**Authors:** John Cagle, David Roth, Cynthia Boyd, Naaz Hussain, Ambrym Smith, Jenni Reiff, Daniel Scerpella

**Affiliations:** University of Maryland, Baltimore, Baltimore, Maryland, United States; Johns Hopkins University, Baltimore, Maryland, United States; Johns Hopkins School of Medicine, Baltimore, Maryland, United States; Johns Hopkins Community Physicians, Baltimore, Maryland, United States; MedStar Union Memorial Hospital, Baltimore, Maryland, United States; Johns Hopkins University, Baltimore, Maryland, United States; Johns Hopkins Bloomberg School of Public Health, Baltimore, Maryland, United States

## Abstract

2. Conducting high-quality advance care planning (ACP) conversations with persons living with ADRD and their family members is inherently challenging. Differing levels of cognitive function, judgment, ability to engage, and care partner involvement adds to the complexity of such ACP conversations, necessitating flexible fidelity monitoring to accommodate contextual factors. This presentation will focus on the process of measuring ACP fidelity. The SHARE ACP fidelity checklist was developed to measure the protocol adherence during ACP meetings, monitor for areas in which ACP facilitators may require re-training or additional support, and to inform analyses of the impact of the overall exposure to the study intervention. The fidelity checklist items are organized into three overarching domains: Meeting Set-Up, ACP Meeting Topics, and Facilitator Communication Skills. For the present study, a fidelity adjudication team from a pool of 7 raters, listened to and independently rated recorded conversations for protocol adherence. Each checklist item is scored ranging from 0 to 2 (0=not done; 1=needs improvement; or 2=effective) with higher scores indicating greater adherence. Fidelity ratings tended to improve over time, suggesting a facilitator experience effect. The goal to achieve an overall fidelity rating of ≥80% was met in 63% of dyad conversations. Based on subscale scores, project targets were most frequently met on the Communication Skills subscale and less frequently accomplished on the Meeting Set Up subscale. Despite some challenges, preliminary assessments of SHARE intervention fidelity appear promising, and evidence suggests the fidelity checklist is both valid and reliable.

